# Spontaneous Pneumothorax Precipitated by a Forceful Sneeze in a Young Male: A Case Report

**DOI:** 10.7759/cureus.104902

**Published:** 2026-03-09

**Authors:** Hüseyin Aldemir

**Affiliations:** 1 Emergency Department, Afyonkarahisar State Hospital, Afyonkarahisar, TUR

**Keywords:** chest tube thoracostomy, forceful sneeze, increased intrathoracic pressure, primary spontaneous pneumothorax, sneeze

## Abstract

Primary spontaneous pneumothorax (PSP) typically affects young, tall, slender males and is often associated with subpleural bleb rupture. While many cases occur at rest, sudden changes in intrathoracic pressure can serve as triggers. A 22-year-old male smoker (BMI: 21.2 kg/m²) presented with acute pleuritic chest pain and dyspnea immediately following a forceful sneeze. Clinical examination and radiography confirmed a massive left-sided pneumothorax. An emergent tube thoracostomy was performed, resulting in immediate lung re-expansion and symptomatic relief. This case highlights the importance of considering PSP in the differential diagnosis of acute chest pain following physiological maneuvers like sneezing, especially in patients with high-risk phenotypes.

## Introduction

Spontaneous pneumothorax (SP) is characterized by the presence of air in the pleural space without an external traumatic or iatrogenic insult [1]. It is categorized into primary SP (PSP), occurring in individuals without known underlying lung disease, and secondary SP (SSP), associated with existing pulmonary pathology such as chronic obstructive pulmonary disease or cystic fibrosis [1-3]. The classic demographic for PSP is young, tall, and thin males [3,4]. The pathophysiological hallmark is the rupture of small, air-filled sacs known as subpleural blebs or bullae, which are usually located at the lung apices [2,4]. While the exact etiology of bleb formation remains debated, smoking and genetic factors are recognized as major contributors [1,2].

Although most episodes occur at rest, abrupt increases in transpulmonary pressure, such as those during coughing, sneezing, or heavy lifting, can precipitate rupture of pre-existing blebs [5-7].The management of SP is tailored to the subtype, size of the collapse, and clinical stability of the patient, ranging from conservative observation and simple aspiration to interventional procedures such as tube thoracostomy and video-assisted thoracoscopic surgery (VATS) [3,4,8].

## Case presentation

**Figure 1:**
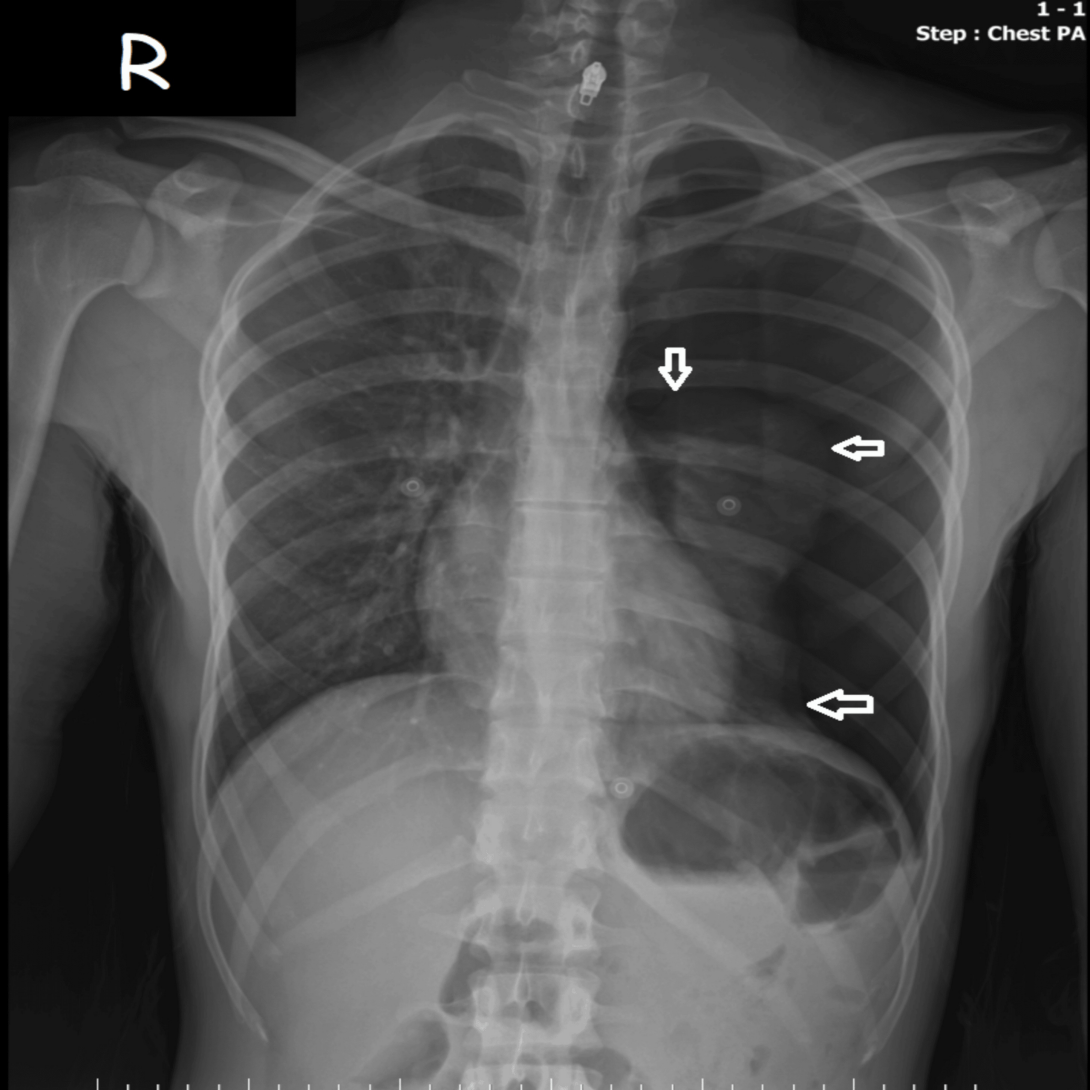
Initial PA chest radiograph showing a large left-sided PSP. White arrows indicate the visceral pleural line (pneumothorax border) with the absence of peripheral lung markings PA: posteroanterior, PSP: primary spontaneous pneumothorax

A 22-year-old male with no significant past medical history presented to the emergency department experiencing an acute onset of sharp, left-sided chest pain and progressive dyspnea immediately following a vigorous sneeze. The patient had no pathology on x-ray during a routine pre-employment examination one year ago, has no known illnesses, and the current complaints began prior to admission. The patient, a chronic cigarette smoker (8 pack-years), exhibited a characteristically asthenic habitus with a height of 188 cm and a weight of 75 kg (BMI: 21.2 kg/m²). Upon clinical examination, the patient was tachypneic, with a respiratory rate of 24 bpm and an oxygen saturation (SpO₂) of 80% on room air, while maintaining a stable blood pressure of 130/90 mmHg and a heart rate of 98 bpm. Physical findings were classic for pneumothorax, including absent breath sounds over the left hemithorax and hyperresonance on percussion. Physical examination revealed significant tracheal deviation to the right, although jugular venous distension was not yet prominent. A standing posteroanterior (PA) chest radiograph (Figure 1) confirmed a large left-sided PSP with near-complete lung collapse and a clearly demarcated visceral pleural line. Crucially, the radiograph demonstrated a distinct mediastinal shift, confirming the clinical finding of tracheal displacement.

**Figure 2:**
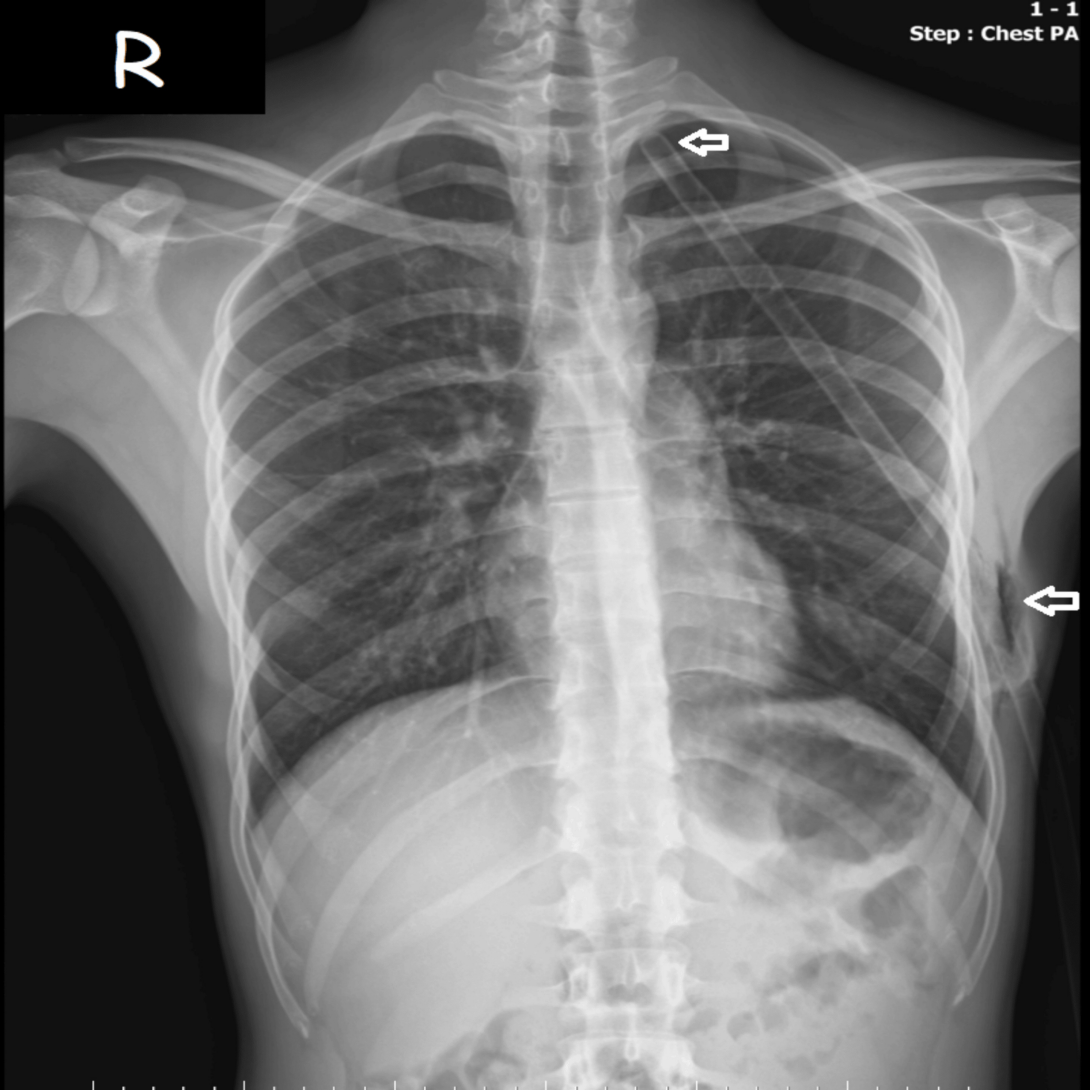
Post-interventional PA chest radiograph demonstrating complete lung re-expansion. The chest tube is visible in the left pleural space, with the insertion site and apical positioning clearly identified. White arrows indicate the chest tube and its insertion site PA: posteroanterior

Following the diagnosis, the patient was immediately started on high-flow supplemental oxygen at 10 L/min. Given the significant degree of lung collapse and clinical respiratory distress, an emergency tube thoracostomy was indicated. A 24 Fr chest tube was successfully inserted at the fifth intercostal space along the midaxillary line. The procedure resulted in an immediate audible rush of air, providing rapid symptomatic relief. A follow-up chest radiograph (Figure 2) was obtained post-procedure, demonstrating successful and complete re-expansion of the left lung.

The patient’s clinical course remained stable; he was initially admitted to the second-level intensive care unit under the thoracic surgery department for close monitoring. Following a period of stability, he was transferred to the thoracic surgery ward. The air leak was resolved by the third day, allowing for the removal of the chest tube. He was subsequently discharged with comprehensive counseling on smoking cessation and the risk of recurrence.

## Discussion

The incidence of PSP is estimated at 18-28 per 100,000 cases per year among males, with a strong predilection for young, tall, and slender individuals [3]. This specific phenotype is associated with a more pronounced pleural pressure gradient from the lung base to the apex, which is thought to facilitate the formation of subpleural blebs or bullae [1,2]. The mechanical trigger in this case, a forceful sneeze, serves as a critical precipitating event. A sneeze generates a massive spike in positive intrathoracic pressure followed by a rapid decline; such a sudden pressure fluctuation can exceed the structural integrity of pre-existing apical blebs, leading to their rupture into the pleural space [5-7]. Recent literature, including reports of sneezing-induced lung herniation, bilateral pneumothorax, and the Macklin effect, suggests that while these physiological reflexes are common, they can act as the final mechanical trigger for the rupture of underlying, pre-existing blebs in susceptible individuals [4-7].

Regarding management, current international guidelines emphasize that drainage is mandatory for large, symptomatic PSP [3,4]. The management strategy is typically tailored to the pneumothorax subtype, the degree of lung collapse, and the patient's clinical stability, encompassing options ranging from conservative observation to interventional procedures such as tube thoracostomy or VATS [3,4,8].

While simple needle aspiration may be considered in smaller or less symptomatic cases, emergency tube thoracostomy remains the definitive "gold standard" for patients presenting with massive collapse or significant hypoxia, as evidenced by this patient’s initial SpO₂ of 80% [3,8]. The absence of systemic hypotension (130/90 mmHg) and stable hemodynamics further categorized this presentation as a non-tension event rather than a tension pneumothorax, allowing for a controlled yet rapid intervention. This case reinforces that even benign physiological reflexes can lead to significant pulmonary morbidity in at-risk populations, necessitating prompt radiological confirmation and decisive mechanical decompression to ensure full recovery.

## Conclusions

SP must remain a high-priority differential diagnosis in young, tall, and lean male smokers who present with acute-onset chest pain and dyspnea, particularly following activities that increase intrathoracic pressure. This case underscores that even routine physiological actions, such as a forceful sneeze, can precipitate significant barotrauma in patients with underlying pulmonary blebs. Stable hemodynamic parameters should not mislead clinicians; the absence of hypotension does not rule out a massive pneumothorax, though it helps differentiate it from a life-threatening tension event. Early clinical suspicion, followed by immediate radiological confirmation and prompt decompression via tube thoracostomy, is essential to mitigate respiratory distress and prevent the potentially fatal progression to tension pneumothorax. Finally, smoking cessation remains the cornerstone of long-term management to reduce the risk of recurrence in this patient population.
